# Clinical Characteristics of Hospitalized Patients With COVID-19: A Retrospective Cohort Study in Pakistan

**DOI:** 10.7759/cureus.44405

**Published:** 2023-08-30

**Authors:** Usman I Aujla, Imran A Syed, Abdullah Khalid, Muhammad Farooq Hanif, Ahmad K Malik

**Affiliations:** 1 Gastroenterology and Hepatology, Pakistan Kidney and Liver Institute and Research Center, Lahore, PAK; 2 Surgery, Pakistan Kidney and Liver Institute and Research Center, Lahore, PAK

**Keywords:** inflammatory markers, comorbidities, disease severity, sars-cov-2, coronavirus disease 2019 (covid-19)

## Abstract

Background: The severe acute respiratory syndrome coronavirus 2 (SARS-CoV-2) caused a global pandemic of severe upper respiratory tract infections known as COVID-19. This single-center study aimed to investigate the demographics, comorbidities, symptoms, and disease severity of COVID-19 patients in Pakistan.

Methods: A retrospective descriptive study was conducted at the Pakistan Kidney and Liver Institute and Research Center from April 2020 to August 2020. A total of 430 PCR-positive COVID-19 patients were categorized into symptomatic and asymptomatic groups. The symptomatic group was further classified into severe and non-severe subgroups. Patients' demographics, comorbid conditions, presenting symptoms, laboratory parameters, and clinical outcomes were assessed in these two subgroups. Statistical tests were applied to determine significant differences.

Results: A total of 430 patients with COVID-19 presented in our center, of whom 334 (78%) were symptomatic and included in the study. Severe disease was observed in 83 (24.8%) symptomatic patients, with a male predominance (75.9%) and higher mean age (61.7 ± 13.2). Travel to high-risk destinations (p < 0.002) and close contact with COVID-19 patients (p < 0.001) were significant risk factors. Major comorbid conditions included diabetes mellitus (30.5%) and hypertension (39.8%). Frequent symptoms included fever (71.8%), cough (68.8%), dyspnea (53.8%), and myalgias (35.9%). Higher C-reactive protein (median = 12.76 vs. 1.45, p = 0.001), ferritin (median = 996.70 vs. 628, p = 0.004), and D-dimers (median = 1121 vs. 439.50, p = 0.009) were noted in severe vs non-severe disease. Lymphopenia was more prevalent in severe vs. non-severe disease (83.1% vs. 14.3% p-value = 0.033). More deaths (28.9%) and ICU admissions (53%) with a prolonged hospital stay (median = 25 days, IQR = 16.0-31.0) were noted in the severe group.

Conclusion: This retrospective study provides insights into the clinical characteristics and outcomes of COVID-19 patients. Age, male gender, comorbidities, and specific symptoms were associated with disease severity. Inflammatory markers, including D-dimers, ferritin, and CRP, were elevated in severe cases. These findings contribute to a better understanding of COVID-19 and may aid in clinical management and decision-making for patients affected by the disease.

## Introduction

The global pandemic of severe upper respiratory tract infection was caused by severe acute respiratory syndrome coronavirus 2 (SARS-CoV-2) [1]. The number of patients infected with COVID-19 doubled in a week and increased sporadically [2]. It was the high incidence of transmission and death that alarmed medical professionals and researchers. Belonging to the Coronaviridae family, severe acute respiratory syndrome coronavirus-2 (SARS-CoV-2) is a small virus (65-125 nm in diameter), consisting of a single-stranded ribonucleic acid (RNA), ranging from 26 to 32 kbps in length [3]. It was named novel coronavirus-2019 (2019-n COV) by Chinese scientists after its outbreak in Wuhan, China, in 2019, causing more than 1800 deaths and infecting approximately 70,000 people during the first five days of the epidemic. Later, the International Committee on Taxonomy of Viruses renamed it SARS-CoV-2 and its disease was termed COVID-19 [4,5].

Pathogenesis of COVID-19 is similar to a typical respiratory coronavirus in terms of infectivity, replication, and mutations. However, some unique mutations increase its transmissibility and infectivity [6]. Scientists found that SARS-CoV-2 is detectable in aerosols for up to three hours, up to 24 hours on cardboard, and up to two to three days on plastic and stainless steel [7].

Thus, the virus spreads via close contact with an infected person and exposure to aerosols containing the virus while sneezing or coughing [8]. These aerosols can penetrate the lungs via inhalation through the nose or the mouth. It can also spread after touching contaminated objects [9,10].

The clinical spectrum of the infection is broad, ranging from patients being asymptomatic to severe viral pneumonia, respiratory failure, and death [11]. Consequently, COVID-19 infection can be roughly classified into three phases: phase I, an asymptomatic incubation period with or without a detectable viral load; phase II, a non-severe symptomatic period with the detectable virus; and phase III, a severe respiratory symptomatic phase with high viral load [9,12]. The prognosis of the condition was influenced by various other variables, including the patient's age, gender, treatment options, immune status, comorbidities, and subsequent infections [12]. For critical patients, it caused serious concerns among clinicians as they contemplated the best therapy options.

The World Health Organization (WHO), on March 11, 2020, declared the novel coronavirus (COVID-19) outbreak a global pandemic [13]. The coronavirus disease 2019 (COVID-19) pandemic has had a crippling impact on healthcare systems across the globe. By April 2020, approximately 2.5 million had been infected by this deadly virus worldwide, and the reported number of deaths reached up to 177,641 globally [14]. Pakistan also suffered from the debilitating impact of this deadly pandemic [15]. The study aimed to know about patients' demographics, the impact of comorbidities on severity, the spectrum of symptoms at presentation, clinical course after admission, and outcomes of the disease. Various inflammatory markers and their association with disease severity were also assessed.

## Materials and methods

It was a retrospective descriptive study conducted from April 2020 to August 2020 at the Pakistan Kidney and Liver Institute and Research Center, Lahore, Pakistan. Only PCR-positive COVID-19 patients were included in the study regardless of age, gender, and comorbidities. A total of 430 patients with positive COVID-19 PCR presented to us during the study period. Based on their history, they were further divided into symptomatic (n=334) and asymptomatic groups (n=96). The symptomatic group was further classified into severe and non-severe groups. This classification was based on their oxygen saturation and respiratory rate (RR). Patients with a consistent oxygen saturation of ≥ 94% and a RR < 25 breaths/minute were sub-grouped into the non-severe category. On the other hand, patients with an oxygen saturation of < 94% and RR ≥ 25 breaths/minute were put into the severe classification. The severe group also included those patients who required noninvasive ventilation (NIV), a high-flow nasal cannula (HFNC), or mechanical ventilation (MV) to maintain their oxygen saturation. After stratifying symptomatic patients according to these two groups, we compared various parameters in both the severe and non-severe groups. Asymptomatic patients were not included in the analysis. The study was approved by the Institutional Review Board (IRB) of the Pakistan Kidney and Liver Institute and Research Center (PKLI&RC), Lahore, Pakistan (IRB Approval Number: PKLI-IRB/AP/023).

The study analyzed the electronic medical records of 430 COVID-19 patients admitted at PKLI&RC, Pakistan. Data were collected from the electronic medical records of the institute. Only patients with confirmed COVID-19 by PCR were admitted. We recorded the possible history of exposure, signs and symptoms, and laboratory findings at admission. Radiologic assessments included chest radiography or high-resolution computed tomography (HRCT), and all laboratory tests were performed according to the hospital-based guidelines. Laboratory investigations included a complete blood count, coagulation profile, assessment of liver and renal functions, serum electrolytes, C-reactive protein, procalcitonin (where needed), lactate dehydrogenase, serum ferritin, and D-dimers.

Data were analyzed with Statistical Package for Social Sciences (SPSS) version 23 (IBM Corp., Armonk, NY). Quantitative variables were presented as median with an interquartile range (IQR) or as means with the standard deviation (SD). Qualitative variables were presented in frequency and percentages. The chi-square test was used to control the qualitative variables, and an independent t-test was applied to make significant differences between the two groups, i.e., severe and non-severe. A p-value of 0.05 was considered statistically significant.

## Results

Among the symptomatic patients mean age was higher (61.7 ± 13.2) for severe disease. Male predominance was noted in both groups. The male-to-female ratio was 3:1 in the severe vs. 1.97:1 in the non-severe group (Table 1).

**Table 1:** Demographic distribution among the symptomatic group.

Demographic characteristics	Total Symptomatic (n= 334)	Non-Severe (n=251)	Severe (n=83)	p-value
Age Mean (Range)	47.3 ± 16.5 (11-93)	45.9 ± 15.3 (14-87)	61.7 ± 13.2 (33-93)	0.001
Male	229 (68.6%)	166 (66.4%)	63 (75.9%)	0.05
Female	105 (31.4%)	84 (33.6%)	21 (24.1%)	

A total of 430 patients diagnosed with COVID-19 presented during the study period. Out of these, 77.7% (n=334) patients were symptomatic and included in the study. Among symptomatic patients, severe disease was observed in 24.8% (n=83), and non-severe illness was observed in 75.15% (n=251) of patients. Close contacts with confirmed COVID-19 and travel to high-risk destinations (regions with a high prevalence of COVID-19, including China, most countries in Europe, as well as some cities in Pakistan with a heavy burden of disease) were identifiable risk factors in this cohort. All healthcare workers detected with COVID-19 (n=59) were symptomatic, most falling into the non-severe (n=51) category (Table 2).

**Table 2:** Risk factors associated with disease severity.

Risk factor	Total n (%)	Non-Severe n (%)	Severe n (%)	p-value
Health care workers	59 (17.7%)	51 (20.3%)	8 (9.6%)	0.091
Close Contact with Confirmed COVID-19 Case	95 (28.4%)	31 (12.3%)	64 (77.1%)	0.001
Travel to a high-risk destination	39 (11.7%)	15 (5.9%)	24 (28.9%)	0.002

Comorbidities were common in both severe and non-severe groups. Diabetes mellitus, hypertension, ischemic heart disease, heart failure, cerebrovascular accident, and autoimmune diseases were statistically significant, with a higher percentage in the severe group (Table 3).

**Table 3:** Distribution of COVID-19-positive patients according to comorbidities. HTN: hypertension, DM: diabetes mellitus; IHD: ischemic heart disease; CKD: chronic kidney disease; CCF: congestive cardiac failure; CVA: cerebrovascular accident

Variables	Total n (%)	Non-Severe n (%)	Severe n (%)	P-Value
HTN	133 (39.8%)	80 (31.8%)	49 (59.0%)	0.001
DM	102 (30.5%)	60 (23.9%)	37 (44.6%)	0.001
IHD	39 (11.6%)	21 (8.4%)	18 (21.7%)	0.001
CKD	27 (8%)	17 (6.7%)	10 (12.0%)	0.086
CCF	2 (0.5%)	0 (0%)	2 (2.4%)	0.014
CVA	6 (1.7%)	2 (0.8%)	4 (4.8%)	0.017
Malignancy	1 (0.3%)	1 (0.4%)	0 (0%)	0.565
Asthma	17 (5.0%)	9 (3.6%)	7 (8.4%)	0.073
Hepatitis B	6 (1.7%)	1 (0.4%)	2 (2.4%)	0.092
Hepatitis C	5 (1.5%)	3 (1.2%)	2 (2.4%)	0.430
Autoimmune Diseases	4 (1.2%)	1 (0.4%)	3 (3.6%)	0.020
Organ Transplantation	2 (0.6%)	2 (0.8%)	0 (0%)	0.415

A broad spectrum of symptoms was observed among the symptomatic group, but fever, cough, dyspnea, and myalgias were the most frequently occurring (Table 4).

**Table 4:** Distribution according to symptoms.

Variables	Total n (%)	Non-Severe n (%)	Severe n (%)	P-value
Fever	240 (71.8%)	172 (68.5%)	68 (82.0%)	0.014
Cough	230 (68.8%)	168 (66.9%)	62 (74.6%)	0.165
Dyspnea	180 (53.8%)	105 (41.8%)	75 (90.4%)	0.001
Sputum Production	62 (18.6%)	43 (17.1%)	19 (22.9%)	0.242
Sore Throat	69 (20.6%)	56 (22.3%)	13 (15.7%)	0.195
Hemoptysis	10 (2.9%)	6 (2.3%)	4 (4.8%)	0.260
Chills N Rigors	13 (3.9%)	9 (3.5%)	4 (4.8%)	0.614
Headache	27 (8.1%)	25 (9.9%)	2 (2.4%)	0.029
Fatigue	42 (12.6%)	29 (11.5%)	13 (15.7%)	0.328
Myalgias	120 (35.9%)	94 (37.5%)	26 (31.3%)	0.313
Arthralgias	10 (2.9%)	5 (1.9%)	5 (6.02%)	0.031
Nausea	20 (5.9%)	18 (7.1%)	2 (2.4%)	0.113
Vomiting	9 (2.7%)	7 (2.7%)	2 (2.4%)	0.853
Diarrhea	49 (14.6%)	42 (16.7%)	7 (8.4%)	0.064
Abdominal Pain	14 (4.2%)	12 (4.7%)	2 (2.4%)	0.350
Conjunctival Congestion	1(0.3%)	1 (0.4%)	0 (0%)	0.565
Nasal Congestion	5 (1.5%)	5 (1.9%)	0 (0%)	0.195
Chest Tightness	28 (8.4%)	19 (7.5%)	9 (10.8%)	0.351
Anosmia	8 (2.4%)	8 (3.1%)	0 (0%)	0.100
Ageusia	5 (1.5%)	5 (1.9%)	0 (0%)	0.195
Dyspepsia	13 (3.9%)	13 (5.1%)	0 (0%)	0.034
Anxiety	26 (7.8%)	21 (8.3%)	5 (6.0%)	0.490
Anorexia	12 (3.6%)	9 (3.5%)	3 (3.6%)	0.990
Flue	22 (6.6%)	17 (6.7%)	5 (6.0%)	0.812

Median values and IQR for various inflammatory markers were calculated, and significantly higher d-dimers, ferritin, and CRP values were observed among the severe group. In contrast, changes in LDH, procalcitonin, and troponin were not statistically significant (Table 5).

**Table 5:** Inflammatory markers among severe and non-severe groups.

Variables	Non-Severe Median (Range)	Severe Median (Range)	P-Value
D-dimer(ng/ml)	n=131; 439.50 (199.50-1120.0)	n =73; 1121 (570-3034)	0.009
C-reactive protein (mg/dl)	n =227; 1.45 (0.25-6.34)	n =79; 12.76 (5.72-21.02)	0.001
Ferritin (ng/ml)	n =145; 628 (279.43-1287.43)	n =77; 996.70 (583.6-2312.5)	0.004
Lactate dehydrogenase (U/L)	n =30; 304 (215-402.25)	n =52; 472 (347-600)	0.151
Troponin (ng/ml)	n =15; 0.01 (0.01-0.10)	n =33; 0.1600 (0.01-0.195)	0.649
Procalcitonin (ng/L)	n =23; 0.102 (0.030-0.250)	n =39; 0.308 (0.113-0.463)	0.181

Median and IQR values for other laboratory investigations were calculated and observed. The following lab tests were statistically significant: AST, GGT, and lymphocytes less than 1 x10^9/L (Table 6).

**Table 6:** Laboratory findings. ALT: alanine transaminase; AST: aspartate aminotransferase; ALP: alkaline phosphatase; GGT: gamma-glutamyl transferase; Hb: hemoglobin; WCC: white cell count

Variables	Total n =334	Non-Severe n (251)	Severe n (83)	p-value
ALT (U/L) Median (IQR)	38.0 (24.0-62.0)	39.0 (26.0-63.0)	43.0 (31.0-64.0)	0.090
AST (U/L) Median (IQR)	32.0 (22.0-52.0)	32.0 (23.0-51.0)	44.0 (30.0-60.0)	0.020
ALP (U/L) Median (IQR)	71.0 (56.0-94.25)	70.0 (57.0-94.75)	83.0 (63.0-98.0)	0.945
Bilirubin (mg/dl) Median (IQR)	1.0 (0.0-1.0)	1.00 (0.00-1.00)	1.00 (0.00-1.00)	0.663
GGT (U/L) Median (IQR)	55.0 (32.0-101.25)	53.0 (34.2-96.75)	78.0 (48.0-134.0)	0.004
WCC (10 x 9/L) Median (IQR)	8.22 (6.20-10.97)	7.57 (6.04-9.78)	12.50 (9.37-15.55)	0.338
Lymphocyte (10x 9/L) Median (IQR)	1.58 (0.97-2.27)	1.96 (1.40-2.57)	0.95 (0.60-1.30)	0.184
Lymphocyte <1 (10x9/L)	105 (31.43%)	36 (14.3%)	69 (83.13%)	0.033
Platelets (10 x 9/L) Median (IQR)	257.0 (197.2-339.1)	258.0 (207.0-333.0)	282.50 (219.0-358.63)	0.641
Platelets<150 (10 x 9/L)	42 (12.5%)	19 (7.56%)	23 (27.71%)	0.251
Hb (g / dl) Median (IQR)	13.60(11.80-14.70)	13.90 (12.53-14.90)	13.40 (11.86-14.20)	0.585
Urea (mg/dl) Median (IQR)	33.80 (22.0-51.85)	28.50 (21.95-43.00)	55.20 (40.38-78.21)	0.296
Creatinine (mg/dl) Median (IQR)	0.827 (0.720-1.032)	0.83 (0.75-1.03)	0.83 (0.77-1.28)	0.288

Various pharmacological and non-pharmacological interventions were executed based on symptoms and disease progression. Results are shown as frequencies in the table below. Higher percentages were noted among the severe group. Admission in the ICU and mortality frequency were higher among severe cases, whereas the recovery rate was higher in the non-severe group. A more prolonged hospital stay was noted among patients suffering from severe disease (Table 7).

**Table 7:** Comparison of treatment and outcome variables.

Variables	Total n (%)	Non-Severe n (%)	Severe n (%)	P-Value
Systemic Glucocorticoids	149 (44.6%)	72 (28.6%)	77 (92.7%)	0.001
Antibiotics (Azithromycin, Moxifloxacin, Carbapenems)	194 (58.1%)	140 (55.7%)	54 (65%)	0.001
Anti-Coagulants	162 (48.5%)	103 (41%)	59 (71%)	0.001
Antifungals (Fluconazole, Voriconazole, Nystatin)	65 (19.5%)	6 (2.3%)	59 (71.0%)	0.001
Oxygen Therapy (≤5L)	137 (41.0%)	85 (33.9%)	52 (62.6%)	0.001
Mechanical Ventilation	56 (16.8%)	31 (12.4%)	25 (30.1%)	0.001
Noninvasive Ventilation	24 (7.2%)	11 (4.4%)	13 (15.6%)	0.001
Tocilizumab	44 (13.17%)	15 (5.9%)	29 (34.9%)	0.001
Intravenous Immunoglobulin	6 (1.8%)	1 (0.4%)	5 (6.0%)	0.001
Convalescent Plasma	4 (1.2%)	1 (0.4%)	3 (3.6%)	0.005
ICU Admission	66 (19.7%)	22 (8.7%)	44 (53%)	0.002
Deaths	26(7.8%)	2 (0.8%)	24 (28.9%)	0.001
Recovered	202 (60.4%)	167 (66.5%)	35 (42.2%)	0.001
Days to Discharge	19.0 (14.0-25.0)	18.0 (14.0-23.0)	25.0 (16.0-31.0)	0.002

## Discussion

This study represents the dynamics of the first wave of COVID-19 in Pakistan. The daily death toll during the first wave touched around 120-130 cases per day. The total number of new cases during this study period across Pakistan was 296,149, and the total number of deaths reported was 6,298, with a death rate of 2.12% [12]. In our study death rate was around 6%. Our cohort's slightly higher death rate was due to selection bias as PKLI&RC was declared a dedicated COVID-19 healthcare facility receiving rather complex referrals.

In our cohort, 24.7% of the patients experienced severe disease. As expected, the death rate was much higher among cases of severe disease (28.9%) vs non-severe disease (0.8%). A study from India also reported higher mortality rates among patients severely affected by COVID-19 [16].

Patients with severe disease were predominantly males and older, having multiple comorbid conditions, including diabetes mellitus, hypertension, ischemic heart disease, congestive cardiac failure, and cerebrovascular and autoimmune diseases. In our study, close contact with confirmed COVID-19 patients and travel to high-risk destinations were the identifiable risk factors for disease transmission.

A meta-analysis in Korea and a study by Rashedi et al. from Iran identified some environmental and host factors that contributed to the spread of disease. Amongst the host factors, old age and comorbid conditions like diabetes mellitus, hypertension, cardiovascular diseases, and malignancy were significant risk factors. Some environmental factors like close contact with COVID-19 patients resulted in rapid viral spread through coughing, sneezing, talking loudly, and contact with contaminated objects [17].

The most frequent presenting symptoms in our cohort were fever (71.85%), cough (68.86%), dyspnea (53.89%), and myalgias (35.92%). Other studies have reported these symptoms as the most frequent presenting complaints [9]. In comparison with the non-severe group, patients suffering from severe COVID-19 disease experienced a significantly higher frequency of fever (82% vs. 68%), dyspnea (90% vs. 41%), and cough (74% vs. 66%).

Among laboratory parameters significant rise in CRP (median = 12.76 vs. 1.45, p= 0.001), ferritin (median = 997 vs. 627, p=0.004), and d-dimers (median = 1121 vs. 440, p=0.009) was noted in severe vs. non-severe disease. Elevation of these markers has been reported as a predictor of severity and mortality in various studies [16]. Lymphopenia was more pronounced in severe vs. non-severe disease (83.3% vs. 14.2% p-value = 0.003).

Most patients (58.05%) received antibiotics, and 13.2% received tocilizumab. Antifungals were used in 19.5% of patients. Oxygen therapy (up to 5 liters) was given to 41.0% of patients, whereas mechanical ventilation was utilized frequently among severe vs. non-severe groups (30.1% vs. 12.4 %). Similarly, the utility of noninvasive ventilation was higher among severe cases (15.7%) vs. non-severe (3.2%), as supported by other studies [18,19]. Our mortality rate of 28.9%, was similar to other studies, including a case series analysis by Richardson et al. (24.5%) [20].

Some limitations of this study should be acknowledged. Firstly, the study focused only on patients admitted to a single center, which may limit the generalizability of the findings to the entire Pakistani population. The study also did not explore the long-term effects and complications of COVID-19 in its population. Despite these limitations, this study provides valuable insights into the clinical characteristics and risk factors associated with severe COVID-19 in Pakistan, contributing to our understanding of the disease and informing future research and public health interventions.

## Conclusions

In conclusion, this retrospective cohort study aimed to investigate the clinical characteristics of hospitalized patients with COVID-19 in Pakistan during the first wave of the pandemic. The findings revealed that severe disease was observed in many symptomatic patients, with a higher risk among older individuals and those with comorbidities. Travel to high-risk destinations and close contact with COVID-19 patients were identified as significant risk factors. Common symptoms included fever, cough, dyspnea, and myalgias. Laboratory parameters such as elevated C-reactive protein, ferritin, D-dimers, and lymphopenia were associated with severe disease. The severe group had higher mortality rates and more frequent ICU admissions with longer stays. These results highlight the importance of age, comorbidities, and specific laboratory markers as predictors of severe COVID-19 disease. The findings contribute to understanding the disease dynamics and can inform clinical management and resource allocation during future waves of the pandemic. Further research is warranted to explore the evolving nature of the virus and its impact on different populations.
